# Type 2 Diabetes: Also a “Clock Matter”?

**DOI:** 10.3390/nu15061427

**Published:** 2023-03-16

**Authors:** Annamaria Docimo, Ludovica Verde, Luigi Barrea, Claudia Vetrani, Pasqualina Memoli, Giacomo Accardo, Caterina Colella, Gabriella Nosso, Marcello Orio, Andrea Renzullo, Silvia Savastano, Annamaria Colao, Giovanna Muscogiuri

**Affiliations:** 1 Department of Clinical Medicine and Surgery, Section of Endocrinology, Diabetology and Andrology, University of Federico II, 80131 Naples, Italy; 2 Department of Public Health, University of Federico II, 80131 Naples, Italy; 3 Dipartimento di Scienze Umanistiche, Università Telematica Pegaso, Via Porzio, Centro Direzionale, Isola F2, 80143 Naples, Italy; 4 CAD Poliambulatorio Pastena, Distretto 66 ASL Salerno, 84125 Salerno, Italy; 5 Unità Operativa Assistenza Sanitaria Benevento, ASL Benevento, 82100 Benevento, Italy; 6 Distretto 58 ASL Napoli 3 Sud, 80050 Naples, Italy; 7 CAD Via Attilio Barbarulo 86, Distretto 60 ASL Salerno, 84014 Nocera Inferiore, Italy; 8 Cattedra Unesco “Educazione alla Salute e allo Sviluppo Sostenibile”, University of Federico II, 80131 Naples, Italy

**Keywords:** chronotype, type 2 diabetes, glycemic control, HbA1c, BMI

## Abstract

Background: We investigated whether chronotype is associated with glycemic control, antidiabetic treatment, and risk of developing complications in patients with type 2 diabetes (T2DM). Methods: The diabetologists filled out an online questionnaire on the Google Form platform to collect the following parameters of subjects with T2DM: body mass index (BMI), fasting plasma glucose (FPG), glycosylated hemoglobin (HbA1c), diabetes history, antidiabetic treatment, diabetic complications, and chronotype categories. Results: We enrolled 106 subjects with T2DM (M/F: 58/48; age: 63.3 ± 10.4 years; BMI: 28.8 ± 4.9 kg/m^2^). Thirty-five point eight% of the subjects showed a morning chronotype (MC), 47.2% an intermediate chronotype (IC), and 17% an evening chronotype (EC). EC subjects reported significantly higher HbA1c (*p* < 0.001) and FPG (*p =* 0.004) values, and higher prevalence of cardiovascular complications (CVC) (*p =* 0.028) and of subjects taking basal (*p* < 0.001) and rapid insulin (*p =* 0.01) compared to MC subjects. EC subjects reported significantly higher HbA1c (*p* < 0.001) and FPG (*p =* 0.015) than IC subjects. An inverse association was found between chronotype score, HbA1c (r = −0.459; *p* < 0.001), and FPG (r = −0.269; *p =* 0.05), remaining significant also after adjustment for BMI, age, and disease duration. Conclusions: EC is associated with higher prevalence of CVC and poorer glycemic control independently of BMI and disease duration in subjects with T2DM.

## 1. Introduction

Chronotype represents the behavioral expression of the circadian rhythm of a subject [1]. It is possible to classify the patients in three categories: morning chronotype or “lark”, if the subject is more active during the first part of the day, evening chronotype or “owl”, if the subject is more active during the last part of the day, and intermediate chronotype, if the subject is in an intermediate position between morning chronotype and evening chronotype subjects [1].

It has been demonstrated that evening chronotype subjects have more risk of developing type 2 diabetes mellitus, metabolic syndrome, and cardiovascular diseases, compared to morning chronotype subjects [2].

Moreover, a lower chronotype score in the Morningness–Eveningness Questionnaire (MEQ), which indicates an evening chronotype subject, has been inversely associated to body mass index (BMI) values [3] and directly associated to adherence to the Mediterranean diet (MD) [4].

These results could be partially explained by the fact that morning chronotype subjects tend to be more regular concerning the eating habits [5] and more physically active [6].

Furthermore, staying awake at night is associated to a higher frequency of night eating syndrome and a higher consumption of junk food [7]. In general, evening chronotype subjects are characterized by an unhealthy lifestyle, showing increased smoking and alcohol intake rates [8].

Evening chronotype subjects more often experience a worsened sleep quality expressed in terms of a higher Pittsburgh Sleep Quality Index (PSQI) [9], which, in turn, causes an increased caloric consumption, in part because of the energy expenditure due to the extended wakefulness [10].

It has already been demonstrated that evening chronotype subjects with poor quality of sleep tend to have a higher rate of insulin resistance (evaluated by HOMA index) and an increased post prandial glycemia, rather than morning and intermediate chronotype subjects [11]. In turn, short-duration sleepers are at higher risk of developing type 2 diabetes mellitus and impaired glucose tolerance (IGT). This was highlighted by Chaput et al. in 2007, who classified 740 patients among short, normal, or long sleepers groups based on oral glucose tolerance test (OGTT) response [12].

As demonstrated by Anothaisintawee et al. in 2017 in 1014 non-shift workers with prediabetes, evening chronotype is in fact associated with higher values of HbA1c [13].

Other environmental factors need to be taken into account when considering the impact on HbA1c in patients affected by type 2 diabetes mellitus, such as light and noise exposure, which indirectly influences chronotype categories, and walkability of the neighborhood [14]. 

Since being an evening chronotype subject is a risk factor of developing type 2 diabetes mellitus, it is reasonable to hypothesize that chronotype could also have a role in metabolic control in subjects that are already diagnosed with type 2 diabetes mellitus. Indeed, we aim to investigate the association of chronotype categories with metabolic control, the risk of developing diabetic-related complications, and treatment in subjects with type 2 diabetes mellitus.

## 2. Materials and Methods

The participants of this study were adults aged 18 years and above with diagnosis of type 2 diabetes mellitus. The data were collected using an online questionnaire on the Google Form platform, which was filled out by the diabetologists, based on the data of their patients.

The cross-sectional study was performed according to the ethical standards of the institutional and national research committee and to the Declaration of Helsinki. An informed consent was collected from all the study participants.

### 2.1. On Line Questionnaire

Questions from 1 to 13 included the clinical parameters: gender (male/female), age (years), height (cm), weight (kg), type 2 diabetes mellitus duration (years), fasting blood glucose (mg/dL), HbA1c (%), comorbidities (arterial hypertension, dyslipidemia, and coronary heart disease), and diabetic complications (diabetic nephropathy, diabetic neuropathy, and diabetic retinopathy). 

The questions from 14 to 21 were about the diabetic treatment (metformin; sodium glucose cotransporter inhibitors, SGLT-2i; glucagon-like peptide-1 receptor agonists, GLP1-RA; pioglitazone; dipeptidyl peptidase-4 inhibitor, DDP-4i; acarbose; rapid insulin and long-acting insulin).

Questions about comorbidities, complications, and diabetic treatment were answered in a dichotomous format “yes” and “no” in the questionnaire.

The chronotype was assessed through the MEQ, which has 19 questions about the subject’s favorite time to carry out habitual both physical and mental activities. This is a multiple choice questionnaire and it is a 4–5-point numerical scale. The MEQ score has a range from 16 to 86, in which a higher result indicates a more MC subject and vice versa. The 22° question indicated the chronotype score of the patient.

The patients were then divided into three groups: morning chronotype subjects (“lark”: 59–86 score), intermediate chronotype subjects (42–58 score), and evening chronotype subjects (“owl”: 16–41 score).

### 2.2. Clinical Parameters

The anthropometric measures (weight and height) were assessed with light clothes on and no shoes, and the BMI (weight (kg) divided by height squared (m^2^), kg/m^2^) was calculated.

Body weight was determined to the nearest 0.1 kg while using a calibrated balance beam scale (Seca 711; Seca, Hamburg, Germany). BMI was classified according to the World Health Organization’s criteria with normal weight: 18.5–24.9 kg/m^2^; overweight, 25.0–29.9 kg/ m^2^; grade I obesity, 30.0–34.9 kg/ m^2^; grade II obesity, 35.0–39.9 kg/m^2^; and grade III obesity, ≥40.0 kg/m^2^.

The information about comorbidities (arterial hypertension, dyslipidemia, and coronary heart disease), diabetic complications (diabetic nephropathy, diabetic neuropathy, and diabetic retinopathy), and the antidiabetic drugs were collected. According the guidelines of Italian Association for the study of diabetes (SID) [15], the patients were classified into three groups: first-line treatment (metformin), second-line treatment (SGLT-2i, GLP1-RA), and third-line treatment (pioglitazone; DDP-4i; acarbose; insulin).

The glycemic control was assessed by evaluating the fasting blood glucose (mg/dL) and the glycated hemoglobin (HbA1c) (%).

The methodology used for assessing the fasting blood glucose was the hexokinase and glucose-6-phosphate dehydrogenase (G6PDH), while, for the HbA1c, high-performance liquid chromatography (HPLC) was employed. The methodologies applied were the same in all the centers included in the study.

### 2.3. Statistics

SPSS software (PASW version 21.0, SPSS Inc., Chicago, IL, USA) was employed to analyze the collected data.

Results have been described as mean ± standard deviation (SD) or number (%). 

Differences in multiple groups (gender; BMI class; presence of arterial hypertension, dyslipidemia, coronary heart disease, diabetic nephropathy, diabetic neuropathy, and diabetic retinopathy; patients in treatment with metformin, SGLT-2i, GLP1-RA, pioglitazone, DDP-4i, acarbose, rapid insulin, and long-acting insulin; and line of treatment) among the 3 chronotype classes were assessed by the chi-square test.

Differences in the mean values (average age, BMI, type 2 diabetes mellitus duration, fasting blood glucose, and HbA1c) between the three chronotype groups were analyzed by ANOVA test followed by the Bonferroni post-hoc test.

Pearson correlation was employed to evaluate the correlation between chronotype score and age, BMI, type 2 diabetes mellitus duration, fasting blood glucose, and HbA1c.

Linear regression was used to investigate the association of HbA1c and fasting blood glucose, with chronotype controlling for age, BMI, and type 2 diabetes mellitus.

## 3. Results

### 3.1. Descriptive Statistics

One-hundred and six patients were enrolled (age: 63.3 ± 10.4 years; weight: 81.9 ± 16.7 kg; BMI: 28.8 ± 4.9 kg/m^2^). They were fairly distributed for gender: 48 females (45.3%) and 58 males (54.7%).

A total of 20.8% of them showed a normal weight, while 45.3% were overweight, 21.7% suffered from grade I obesity, 9.4% from grade II obesity, and 2.8% from grade III obesity.

Concerning type 2 diabetes mellitus, the mean duration was 10.3 ± 8.2 years; the average fasting blood glucose and HbA1c values were 149 ± 49 mg/dL and 7.5 ± 1.5 %, respectively.

A total of 77.4% of the patients were affected by arterial hypertension, 65.1% by dyslipidemia, and 23.6% by coronary heart disease.

Some of the patients had already developed diabetic complications. In particular, 11.3% of them suffered from diabetic retinopathy, 11.3% from diabetic neuropathy, and 21.7% from diabetic nephropathy.

The diabetic treatment in 83% of the cases included metformin, while 34% included GLP1-RA, 37.7% included SGLT-2i, 23.6% included long-acting insulin, 10.4% included rapid insulin, 7.5% included DDP-4i, 3.8% included pioglitazone, and only 0.9% included acarbose.

For 15.1% of the participants, a first-line treatment was performed, whereas 49.1% of them were following a second-line treatment and 35.8% were following a third-line treatment.

The mean chronotype score was 56 ± 14 and the results showed 17% of evening chronotype subjects, 47.2% of intermediate chronotype subjects, and 35.8% of morning chronotype subjects.

### 3.2. Comparison between Chronotype Categories

Morning chronotype subjects were 44.7% females and 55.3% males, with a mean age of 60.8 ± 10.3 years and a mean BMI of 27.6 ± 3.8 kg/m^2^. Intermediate chronotype subjects were 62% females and 38% males, with a mean age of 65 ± 11 years and a mean BMI of 29.3 ± 5.3 kg/m^2^. Evening chronotype subjects were 55.6% females and 44.4% males, with a mean age of 62 ± 8 years and a mean BMI of 29.9 ± 5.3 kg/m^2^.

A total of 23.7% of the morning chronotype subjects had a normal weight, 50% were overweight, 18.4% were affected by grade I obesity, and 7.9% were affected by grade II obesity. None of these subjects were affected by grade III obesity.

A total of 18% of the intermediate chronotype subjects had a normal weight, 46% were overweight, 24% were affected by grade I obesity, 6% were affected by grade II obesity, and 6% were affected by grade III obesity.

A total of 22.2% of the evening chronotype subjects had a normal weight, 33.4% were overweight, 22.2% were affected by grade I obesity, and 22.2% were affected by grade II obesity. None of these subjects were affected by grade III obesity.

No significant difference was found in terms of distribution in the BMI classes between the three groups.

The differences in terms of HbA1c (morning chronotype subjects: 6.8 ± 0.9%; intermediate chronotype subjects: 7.5 ± 1.4%; evening chronotype subjects: 8.9 ± 1.9%) (*p* < 0.001) and fasting blood glucose (morning chronotype subjects: 138 ± 31 mg/dL; intermediate chronotype subjects: 145 ± 48 mg/dL; evening chronotype subjects: 183 ± 71 mg/dL) (*p* = 0.005) between the three groups were significant.

Although there were not statistically significant differences in terms of age and BMI among the three groups, evening chronotype subjects showed HbA1c levels significantly higher (8.9 ± 1.9%) compared to morning chronotype (6.8 ± 0.9%) (*p* < 0.001) and intermediate chronotype subjects (7.5 ± 1.4%) (*p* < 0.001). 

Furthermore, in evening chronotype subjects, fasting blood glucose levels were significantly higher (183 ± 71 mg/dL) when compared to the values of morning chronotype (138 ± 31 mg/dL) (*p =* 0.004) and intermediate chronotype (145 ± 48 mg/dL) (*p =* 0.015) subjects.

No significant differences were demonstrated comparing HbA1c and fasting blood glucose between morning chronotype and intermediate chronotype subjects.

The three groups did not differ for prevalence of dyslipidemia, diabetic retinopathy, neuropathy, and nephropathy.

In fact, morning chronotype subjects had a 60.5% prevalence of dyslipidemia, while intermediate chronotype subjects had a 62% prevalence and evening chronotype subjects had an 83.3% prevalence.

Diabetic retinopathy was present in 7.9% of the morning chronotype subjects, 10% of the intermediate chronotype subjects, and 22.2% of the evening chronotype subjects.

Diabetic neuropathy was present in 7.9% of the morning chronotype subjects, 10% of the intermediate chronotype subjects, and 22.2% of the evening chronotype subjects.

Diabetic nephropathy was present in 13.2% of the morning chronotype subjects, 26% of the intermediate chronotype subjects, and 27.8% of the evening chronotype subjects.

Among the three groups, there was no significant difference in terms of type 2 diabetes mellitus duration. In fact, morning chronotype subjects had a mean duration of 10.7 ± 8.3 years, intermediate chronotype subjects of 10.1 ± 8 years, and evening chronotype subjects of 10.3 ± 9.1 years.

There was no significant difference even in the prevalence of first, second, or third line of antidiabetic treatment. First-line treatment was employed in 15.8% of the morning chronotype subjects, 16% of the intermediate chronotype subjects, and 11.1% of the evening subjects. Second-line treatment was employed in 60.5% of the morning chronotype subjects, 44% of the intermediate chronotype subjects, and 38.9% of the evening subjects.

A higher prevalence of subjects in third line of treatment was found in evening chronotype subjects (50%) compared to morning chronotype (23.7%) and intermediate chronotype (40%) subjects, although this was a trend, and it did not reach statistical significance (*p =* 0.319).

In addition, there were not significant differences among the three groups in terms of the prevalence of non-insulinic antidiabetic treatment.

Metformin was employed in 81.6% of the morning chronotype subjects, 78% of the intermediate chronotype subjects, and 100% of the evening chronotype subjects.

GLP1-RA were employed in 28.9% of the morning chronotype subjects, 36% of the intermediate chronotype subjects, and 38.9% of the evening chronotype subjects.

SGLT-2i were employed in 34.2% of the morning chronotype subjects, 36% of the intermediate chronotype subjects, and 50% of the evening chronotype subjects.

DDP-4i were employed in 13.2% of the morning chronotype subjects, 4% of the intermediate chronotype subjects, and 5.6% of the evening chronotype subjects.

Pioglitazone was employed in 5.3% of the morning chronotype subjects, 4% of the intermediate chronotype subjects, and none of the evening chronotype subjects.

Acarbose was administered to 2.6% of the morning chronotype subjects and to none of the evening and intermediate chronotype subjects.

The three groups showed different prevalence of treatment both with basal insulin (*p* < 0.001) and rapid insulin (*p =* 0.032). In particular, in evening chronotype subjects, there was a significant higher prevalence of subjects taking basal insulin (*p* < 0.001) and rapid insulin (*p =* 0.010) compared to morning chronotype subjects. 

In addition, the comparison between morning chronotype and evening chronotype subjects highlighted a significantly higher rate of arterial hypertension (*p =* 0.031) and coronary heart disease (*p =* 0.028). In fact, 68.4% of the morning chronotype subjects were affected by arterial hypertension, while 94.4% of the evening chronotype subjects were affected by it. A total of 13.2% of the morning chronotype subjects were affected by coronary heart disease and 38.9% of the evening chronotype subjects were affected by it (Table 1).

### 3.3. Correlation Studies

**Figure 1:**
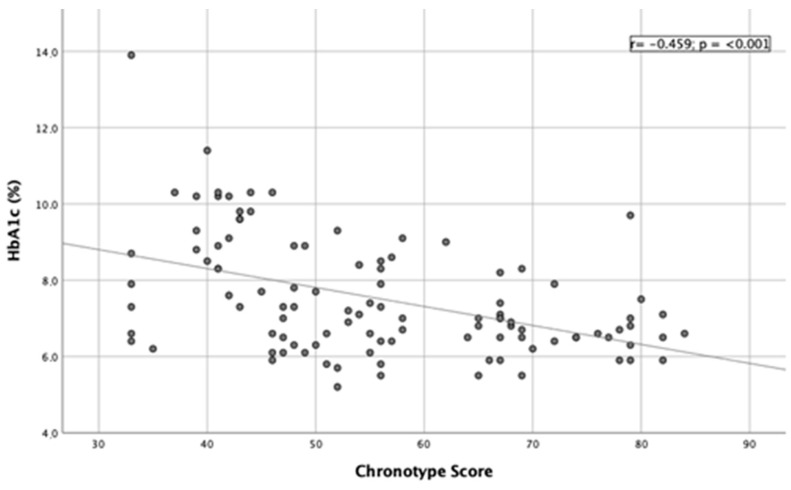
Linear correlation between HbA1c and chronotype score.

**Figure 2:**
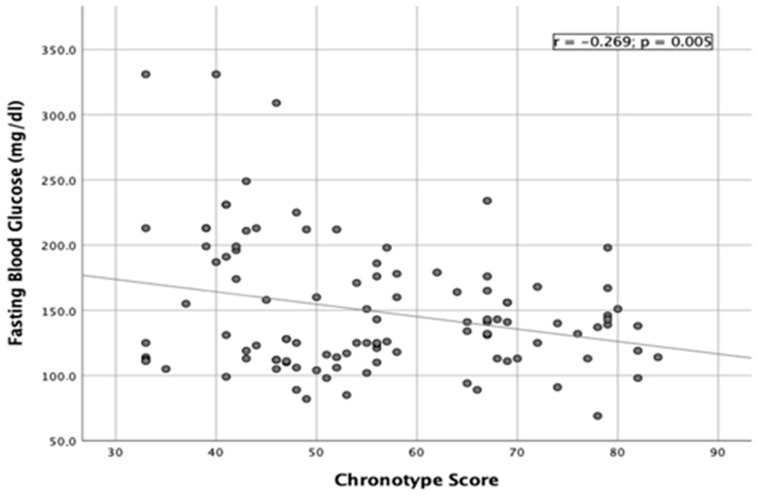
Linear correlation between fasting blood glucose and chronotype score.

A significant inverse correlation was found between chronotype score and HbA1c values (r = −0.459; *p* < 0.001) (Figure 1), showing that a decrease in chronotype score (more evening chronotype subjects) was associated with an increase in HbA1c values (less controlled type 2 diabetes mellitus). An inverse correlation was also found between chronotype score and fasting blood glucose values (r = −0.269; *p =* 0.005) (Figure 2).

This correlation remained significant also correcting the analysis for BMI (*p =* 0.054), age (*p =* 0.112), and type 2 diabetes mellitus duration (*p =* 0.052).

HbA1c was influenced mainly by chronotype score (*p* < 0.001) and less, but still significantly, by BMI (*p =* 0.015) and age (*p =* 0.015), while it was not duration of type 2 diabetes mellitus (*p =* 0.174) (Table 2).

Fasting blood glucose was influenced mainly by chronotype score (*p =* 0.001) and then by age (*p =* 0.002). BMI (*p =* 0.343) and type 2 diabetes mellitus (*p =* 0.190) had no weight on fasting blood glucose (Table 3).

## 4. Discussion

As demonstrated by our study, chronotype could play a role in metabolic control of the type 2 diabetes mellitus, expressed in term of HbA1c and fasting blood glucose. 

In particular, we have demonstrated that evening chronotype subjects had a worse control of type 2 diabetes mellitus. This result was independent from BMI, age, and type 2 diabetes mellitus duration.

In our study, the worse glycemic control of evening chronotype subjects is also demonstrated by the fact that these subjects were more likely in treatment with insulin, both basal and rapid, compared to the morning chronotype subjects. Indeed, in the evening chronotype subjects, there is a trend showing higher prevalence of third line treatment.

Our results are in accordance with the literature, which has already reported that evening chronotype subjects had a worse control of type 2 diabetes mellitus in terms of HbA1c values and the need of insulin treatment [16]. Reutrakul et al. have attributed this finding to the fact that these subjects consumed a higher percentage of the daily calories at dinner compared to the other chronotype categories, and thus experienced a chronodisruption in nutrition. This result was also supported from the evidence that it was independent from the sleep disturbances.

The difference with our study is that they did not use a validated questionnaire such as the MEQ, but they have utilized, as an indicator of chronotype, a construct derived from mid-sleep time on weekends [16]. 

Moreover, Hashemipour et al. demonstrated that the association between evening chronotype subjects and poorer control of type 2 diabetes mellitus is independent from other sleep variables and even from BMI [17].

In addition, von Schnurbein et al., carrying out a study in young patients with type 1 diabetes mellitus, demonstrated the same association between evening chronotype subjects and the need of insulin that we found in our cohort [18].

Although, in our study, subjects belonging to the three categories of chronotype have similar BMI, thus suggesting that they potentially have similar calorie intake, we could hypothesize that evening chronotype subjects were more prone to consume higher percentage of calories at dinner and this could result in a worsened metabolic control as previously reported [19]. In addition, one of the main hormonal determinants of chronotype categories has been identified in cortisol, and evening chronotype subjects have been shown to have a delayed cortisol peak time. Of note, the presence of the cortisol peak at a time of day when it should not be could be responsible for derangements of glucose metabolism. In fact, circadian misalignment and sleep deprivation cause delay on the cortisol rhythm and impairment on the overall exposure to cortisol during the day [20]. In particular, cortisol undergoes a complete inverse pattern across the sleep/wake cycle, peaking after awakening and showing high levels at the end of the wake episode and beginning of the sleep episode, contributing to insulin resistance and hyperglycemia [21].

The circadian disruptions, indeed, cause an inadequate pancreatic beta cell insulin secretion after a standardized meal, explaining the consequent hyperglycemia [22].

Alterations in the cortisol rhythm are associated also with cardiovascular disease [23]. In particular, Muscogiuri et al. in 2021 demonstrated that among 172 middle-aged adults, cardiovascular diseases are more frequent in evening chronotype subjects [3].

In our study, an increased risk of cardiometabolic complications, such as arterial hypertension and coronary heart disease, was highlighted when comparing the evening chronotype subjects with the morning chronotype ones, and this outcome is independent of BMI.

In addition, there are other cross-sectional studies demonstrating an association between chronotype categories and risk of developing type 2 diabetes mellitus [24]. For example, X. Tan et al. in 2020 examined the data from the UK Biobank of 337,083 white British people and an association between evening chronotype subjects and augmented risk of developing type 2 diabetes mellitus was found [24]. In fact, circadian rhythm disruptions are associated with a higher prevalence of hyperphagia, obesity, metabolic syndrome, hyperlipemia, and hyperglycemia, as demonstrated in a study performed in two American and European independent populations by Aguilar-Galarza et al., [25] and insulin-resistance, as proven by Barrea et al. in a 2022 study which investigated 300 women affected by polycystic ovary syndrome [26].

As this is a cross-sectional study, it is not possible to identify the causality and it could be plausible that it is hyperglycemia itself that influences and disrupts the circadian rhythm, causing a desynchronization in metabolically uncompensated patients with type 2 diabetes mellitus. Further studies are needed to evaluate the physiopathological mechanisms which link circadian disruption and poorer glycemic control. In fact, as prolonged and erratic daily eating patterns are associated with a higher risk of developing type 2 diabetes mellitus [27], a targeted dietary therapy for evening chronotype subjects, such as time-restricted eating could be considered to improve the glycemic control of these patients. 

## Figures and Tables

**Table 1:** Comparison between clinical parameters of the three chronotype categories.

Parameters	Morning Chronotype *n* = 38 (35.8%)	Intermediate Chronotype *n* = 50 (47.2%)	Evening Chronotype *n* = 18 (17%)	*p*-Value
Gender				0.272
Female (n, %)	17 (44.7%)	31 (62%)	10 (55.6%)	
Male (n, %)	21 (55.3%)	19 (38%)	8 (44.4%)	
Age	60.8 ± 10.3	65.4 ± 11	62.5 ± 7.8	0.111
Anthropometric measurement BMI (kg/m^2^)	27.6 ± 3.8	29.3 ± 5.3	29.9 ± 5.3	
Normal weight (n, %)	9 (23.7%)	9 (18%)	4 (22.2%)	
Overweight (n, %)	19 (50%)	23 (46%)	6 (33.4%)	
Grade I obesity (n, %)	7 (18.4%)	12 (24%)	4 (22.2%)	0.146
Grade II obesity (n, %)	3 (7.9%)	3 (6%)	4 (22.2%)	0.374
Grade III obesity (n, %)	0 (0%)	3 (6%)	0 (0%)	
Type 2 diabetes mellitus duration (years)	10.7 ± 8.3	10.1 ± 8	10.3 ± 9.1	0.953
Glycemic profile				
Glycemia levels (mg/dL)	138 ± 31 ^b^	145 ± 48 ^a^	183 ± 71 ^a^	0.005
HbA1c (%)	6.8 ± 0.9 ^b^	7.5 ± 1.4 ^a^	8.9 ± 1.9 ^a^	<0.001
Diseases				
Arterial hypertension	26 (68.4%)	39 (78%)	17 (94.4%) ^a^	0.930
Dyslipidemia	23 (60.5%)	31 (62%)	15 (83.3%)	0.202
Retinopathy	3 (7.9%)	5 (10%)	4 (22.2%)	0.264
Neuropathy	3 (7.9%)	5 (10%)	4 (22.2%)	0.264
Nephropathy	5 (13.2%)	13 (26%)	5 (27.8%)	0.277
Coronary heart disease	5 (13.2%)	13 (26%)	7 (38.9%) ^a^	0.91
Treatment				
Metformin	31 (81.6%)	39 (78%)	18 (100%)	0.99
GLP1-RA	11 (28.9%)	18 (36%)	7 (38.9%)	0.700
SGLT-2i	13 (34.2%)	18 (36%)	9 (50%)	0.492
Basal insulin	1 (2.6%)	16 (32%) ^a^	8 (44.4%) ^a^	<0.001
Rapid insulin	0 (0%)	8 (16%) ^a^	3 (16.7%) ^a^	0.032
DPP4I	5 (13.2%)	2 (4%)	1 (5.6%)	0.257
Pioglitazone	2 (5.3%)	2 (4%)	0 (0%)	0.623
Acarbose	1 (2.6%)	0 (0%)	0 (0%)	0.405
1° line treatment	6 (15.8%)	8 (16%)	2 (11.1%)	0.319
2° line treatment	23 (60.5%)	22 (44%)	7 (38.9%)	
3° line treatment	9 (23.7%)	20 (40%)	9 (50%)	

^a^ *p* < 0.05 vs. morning; ^b^ *p* < 0.05 vs. intermediate.

**Table 2:** Linear regression model of association between HbA1c (as a dependent variable) and age, body mass index, type 2 diabetes mellitus duration, and chronotype score as independent variables.

Parameters	Linear Regression Model				
	Non-Standard Coefficients		Standardized Coefficients		
	T	SE	β	t	*p*-Value
Age	−0.032	0.013	−0.220	−2.483	0.015
Body mass index	0.066	0.027	0.209	2.467	0.015
Type 2 diabetes mellitus duration	0.022	0.016	0.120	1.370	0.174
Chronotype score	−0.050	0.009	−0.460	−5.333	0.000

**Table 3:** Linear regression model of association between fasting blood glucose (as a dependent variable) and age, body mass index, type 2 diabetes mellitus duration, and chronotype score as independent variables.

Parameters	Linear Regression Model				
	Non-Standard Coefficients		Standardized Coefficients		
	T	SE	β	t	*p*-Value
Age	−1.485	0.463	−0.310	−3.203	0.002
Body mass index	0.905	0.949	0.088	0.954	0.343
Type 2 diabetes mellitus duration	0.763	0.578	0.126	1.319	0.190
Chronotype score	−1.087	0.333	−0.307	−3.265	0.001

## Data Availability

The data presented in this study are available on request from the corresponding author.
